# Spreading Control in Two-Layer Multiplex Networks

**DOI:** 10.3390/e22101157

**Published:** 2020-10-15

**Authors:** Roberto Bernal Jaquez, Luis Angel Alarcón Ramos, Alexander Schaum

**Affiliations:** 1 Department of Applied Mathematics and Systems, Universidad Autónoma Metropolitana, Cuajimalpa, Mexico-City 05348, Mexico; rbernal@cua.uam.mx; 2 Postgraduate in Natural Sciences and Engineering, Universidad Autónoma Metropolitana, Cuajimalpa, Mexico-City 05348, Mexico; 3 Chair of Automatic Control, Kiel-University, 24143 Kiel, Germany; alsc@tf.uni-kiel.de

**Keywords:** multilayer complex networks, stability, spreading control

## Abstract

The problem of controlling a spreading process in a two-layer multiplex networks in such a way that the extinction state becomes a global attractor is addressed. The problem is formulated in terms of a Markov-chain based susceptible-infected-susceptible (SIS) dynamics in a complex multilayer network. The stabilization of the extinction state for the nonlinear discrete-time model by means of appropriate adaptation of system parameters like transition rates within layers and between layers is analyzed using a dominant linear dynamics yielding global stability results. An answer is provided for the central question about the essential changes in the step from a single to a multilayer network with respect to stability criteria and the number of nodes that need to be controlled. The results derived rigorously using mathematical analysis are verified using statical evaluations about the number of nodes to be controlled and by simulation studies that illustrate the stability property of the multilayer network induced by appropriate control action.

## 1. Introduction

Multiplex networks are a collection of coupled networks placed in different layers with each layer having the same set of nodes but not necessarily the same topology. Layer interactions are given via counterpart nodes of each network layer. Multilayer networks build key elements in the structure of many modern technological systems including social cyber and computer networks as well as in fundamental natural systems determining the functioning of gene regulation and brain dynamics [1,2,3,4,5,6]. A central advantage in comparison to single-layer networks is that each node can have different states in the different networks. This enables them e.g., to analyze the spreading of information or computational viruses among different social or cyber networks [7], thus enabling the identification, understanding and possibly the manipulation of the corresponding mechanisms associated to each layer and between layers.

Spreading processes in complex networks have attracted recent attention for the purpose of analyzing the intertwined dynamics of epidemics [8,9,10,11,12,13] or information transmission in [14,15,16,17,18]. The control of such problems has to address fundamental questions as (i) which parameters of the system are amenable to manipulation and (ii) which nodes must be actively controlled. The latter question goes in particular in hand with the aim to develop control strategies with minimum need of implementation costs. In multilayer networks the additional question arises if nodes need to be controlled in all layers or just in some of them or maybe only in one single layer, as long as the nodes to be controlled are defined accordingly.

The question of network control has been addressed on one side using classical control theory methods as controllability analysis [19,20,21,22,23,24,25,26] including statistic evaluations of the number of nodes to be controlled in networks of certain structures [11,27,28,29,30,31,32,33]. Given that nonlinear system controllability analysis is much more involved than for linear systems [34] controllability studies are typically focussing on linear models or the linearization about some equilibrium point. Only a few recent studies explicitly considered nonlinear controllability and control design approaches in complex networks [25,26]. It should be mentioned that even though network controllability ensures that a desired state can be reached or stabilized, it does not necessarily guide the way for the design of a decentralized control but typically leads to centralized control strategies. On the other hand, the control of networks has been explicitly addressed using stabilization and stability analysis leading the way to the choice of nodes to be controlled with implicit decentralized parametric control strategies [35,36,37,38]. In particular, the approach followed in [36,37,38] yields global stability assessments by means of the derivation of a global dominant linear dynamics. Furthermore, optimization based approaches for parameter adaptation and node or link removal have been widely discussed, as has been summarized in [39].

In the present study the control of a spreading process in a complex multilayer network is addressed on the basis of the classical Markov-based susceptible-infected-susceptible (SIS) dynamics [40,41,42,43,44] in a multilayer version that has been adapted from [7] in such a way that the unit polytope is an invariant set for the dynamics. Following the global stability analysis and parametric control design studies for SIS processes in homogenous and inhomogeneous single-layer complex networks [36,38] and extensions of it including quarantine [37,45] a decentralized parametric control strategy is developed providing sufficient conditions for global stability of the extinction state without altering the topology of the networks as is suggested in other studies related to adaptive networks [39,46]. Instead of involving computationally expensive optimization procedures, simple analytic measures are provided which can be quickly determined for a given network topology and parameter set. Accordingly, the present result provides (i) a solution to the problem of designing decentralized spreading control strategies with global stability assessment and without huge computational effort, which to the knowledge of the authors is still an open question, and (ii) presents an extension of the approaches in [36,37,38] to the case of two-layer multiplex networks. It turns out that the step from a single layer to a two-layer network allows to clearly identify some of the main challenges when considering multiplex networks. In particular, having in mind the nonlinear dynamics in each network and its non-trivial interplay between networks it is clear from the theory of input-to-state stability [47,48] that it is not sufficient that both nonlinear systems are asymptotically stable for their own but the specific interconnection needs to satisfy some additional, small-gain-like criteria. A sufficient criterion ensuring the asymptotic stability of the complete multiplex networks and its differentiation to the stability criteria for each network on its own is a central result that is derived. Based on this criterion it is highlighted how the number of nodes that need to be controlled changes when the interconnection of two networks is considered. Besides a rigorous mathematical derivation of the results some statistical analysis is provided to show the expected variation in the number of nodes that need to be controlled for some illustrative setups.

The paper is organized as follows: In Section 2 the problem formulation is stated, in Section 3 the system analysis is presented along with the main mathematical results of this work. Control design, a statistical analysis of the number of nodes to be controlled, and simulations to corroborate our results are presented in Section 4 and Section 5, respectively. Finally, conclusions are presented in Section 6.

## 2. Problem Formulation

**Figure 1:**
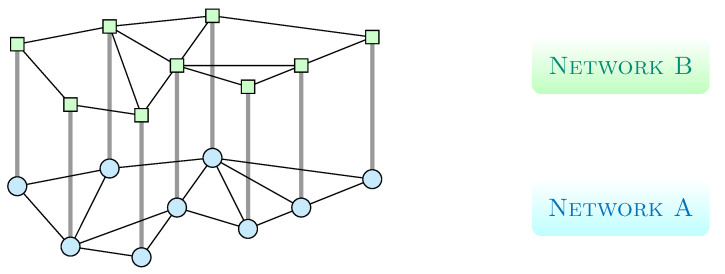
Networks *A* and *B* of arbitrary topology with each node *i* in network *A* being connected with its equivalent node *i* in network *B*.

Consider a two layer network of any topology with adjacency matrices given by A and B. Each network has the same set of *N* nodes, and the adjacency matrix associated to network *A* is defined as $A=[a_{\mathrm{ij}}]$, where $a_{ij}=a_{ji}=1$ if nodes *i* and *j* are connected and zero otherwise (that means, we consider non-directed graphs), the adjacency matrix associated to network *B* is defined in the same way as $B=[b_{\mathrm{ij}}]$. Any node *i* in network *A* is connected with node *i* in the network *B*, as it is shown in Figure 1.

Using a slightly modified version of the model defined in [7,8], the underlying process for every node in both layers of the network is modeled as a discrete time SIS Markov process. A node *i* can be in state *I* (infected) with probability $p_{Ai}\left(t\right)$ (or $p_{Bi}\left(t\right)$) at time $t\in N_{0}$, or in state *S* (susceptible) with probability $1-p_{Ai}\left(t\right)$ (or $1-p_{Bi}\left(t\right)$). The probabilities $p_{Ai}\left(t\right)$ and $p_{Bi}\left(t\right)$ then correspond to the solutions of the following dynamical system:

(1) $$\begin{array}{c} \begin{array}{cc} p_{Ai}\left(t+1\right) & =\left(1-\mu_{Ai}\right)p_{Ai}\left(t\right)+\left(1-q_{Ai}\left(t\right)\right)\left(1-p_{Ai}\left(t\right)\right),\\ p_{Bi}\left(t+1\right) & =\left(1-\mu_{Bi}\right)p_{Bi}\left(t\right)+\left(1-q_{Bi}\left(t\right)\right)\left(1-p_{Bi}\left(t\right)\right),\\ p_{ki}\left(0\right) & =p_{ki0},\;k=\left\{A,B\right\},\;i=1,2,\ldots ,N. \end{array} \end{array}$$

In the preceding Equations $\mu_{ki}$ is the recovery probability of node *i* in the network k∈{A,B}, $q_{ki}\left(t\right)$ is the probability that node *i* in network *k* is not infected by some neighbor in network *A* or *B*, which is given by

(2) $$\begin{array}{cc} q_{Ai} & =\varphi_{Ai}\left(P_{A},P_{B}\right):=\prod_{j=1}^{N}\left(1-\beta_{Ai}a_{ij}p_{Aj}\right)\left(1-\gamma_{Ai}p_{Bi}\right),\\ q_{Bi} & =\varphi_{Bi}\left(P_{A},P_{B}\right):=\prod_{j=1}^{N}\left(1-\beta_{Bi}b_{ij}p_{Bj}\right)\left(1-\gamma_{Bi}p_{Ai}\right), \end{array}$$

with $P_{k}={\left[p_{k1},\ldots ,p_{kN}\right]}^{T}$ for k=A,B. The parameters $\beta_{Ai}$ and $\beta_{Bi}$ represent the transmission probabilities of the node *i* in each layer-network, and $\gamma_{Ai}$ and $\gamma_{Bi}$ are the transmission probabilities of a node *i* from *B* to *A* and from *A* to *B*, respectively.

Note that in Equations (1) and (2)

$$0\leq p_{ki}\left(t\right),\mu_{ki},q_{ki}\left(t\right),\gamma_{ki},\beta_{ki},p_{ki0}\leq 1,\;k=\left\{A,B\right\},\;i=1,2,\ldots ,N.$$

Additionally, in order to propose a control mechanism, we consider that each node has a manipulable variable $u_{ki}\left(t\right)$ (k∈{A,B}), which is amenable for control. In the present study, we consider that the amenable variables are taken from the set $\left\{\gamma_{Ai},\beta_{Ai},\gamma_{Bi},\beta_{Bi};i=1,\ldots ,N\right\}$.

The problem addressed in the following consists in determining the m≤N nodes whose interaction parameters $\gamma_{ki},\beta_{ki}$ have to be adapted in order to ensure the global exponential stability of the extinction state, i.e., such that for all $p_{ki0}\in \left[0,1\right]$ there are constants $m_{ki}\geq 1,\alpha \in \left(0,1\right)$ such that

(3) $$p_{ki}\left(t\right)\leq m_{ki}\alpha^{t}p_{ki0}.$$

## 3. System Analysis

The fixed points $p_{ki}^{*}$, k={A,B}, i=1,…,N associated with the dynamics (1) for some constant values $\mu_{ki}$, $\gamma_{ki}^{*}$, and $\beta_{ki}^{*}$ are determined by substituting $p_{ki}\left(t+1\right)=p_{ki}\left(t\right)=p_{ki}^{*}$ into (1). After some algebra it follows that

(4) $$\begin{array}{c} p_{ki}^{*}=\frac{1-q_{ki}^{*}}{\mu_{ki}+1-q_{ki}^{*}},\;k=\left\{A,B\right\},\;i=1,\ldots ,N,\;q_{ki}^{*}=\varphi_{ki}\left(P_{A}^{*},P_{B}^{*}\right) \end{array}$$

with $\varphi_{ki}$ defined in (2) and $P_{k}^{*}={\left[p_{k1}^{*},\ldots ,p_{kN}^{*}\right]}^{T}$. Note that $p_{ki}^{*}=0$ for all k={A,B},i=1,…,N is a fixed point given that this condition implies that $q_{ki}=1$. This fixed point is referred to as extinction state.

Given that model (1) represents the evolution of probabilities it is important to ensure that all solutions for $p_{ki}$ are contained in the unit hypercube $P={\left[0,1\right]}^{2N}$. This is established in the following Lemma.

**Lemma** **1.** *The set $P={\left[0,1\right]}^{2N}$ is a positively invariant set for the dynamics* (1).

**Proof.**  Let $p_{ki}\left(t\right)\in \left[0,1\right]$, k={A,B}, i=1,…,N. From (1) it follows that

$$\begin{array}{cc} p_{ki}\left(t+1\right) & =\left(1-\mu_{ki}\right)p_{ki}\left(t\right)+\left(1-q_{ki}\left(t\right)\right)\left(1-p_{ki}\left(t\right)\right)\leq p_{ki}\left(t\right)+\left(1-p_{ki}\left(t\right)\right)=1 \end{array}$$

and

$$\begin{array}{cc} p_{ki}\left(t+1\right) & \geq \left(1-\mu_{ki}\right)p_{ki}\left(t\right)\geq 0. \end{array}$$

 □

Next, sufficient conditions for the (global in P) exponential stability of the extinction state ${\left(P_{A},P_{B}\right)}^{T}={\left(0,0\right)}^{T}$ are presented in the following Theorem.

**Theorem** **1.** *Consider the dynamics* (1) *on a two-layer network with adjacency matrices A and B. The extinction state $\left(P_{A},P_{B}\right)=\left(0,0\right)$ is globally exponentially stable in the hypercube P if*

(5) σ(H)<1,

*where σ(·) is the spectral radius, and the matrix H is defined as follows*

$$H=\left[\begin{array}{cc} I-M_{A}+B_{A}A & G_{A}\\ G_{B} & I-M_{B}+B_{B}B \end{array}\right],$$

*where $M_{k}=diag\left(\mu_{ki}\right)$, $B_{k}=diag\left(\beta_{ki}\right)$, $G_{k}=diag\left(\gamma_{ki}\right)$ (k∈{A,B}), and I is the identity matrix.*

**Proof.**  The exponential stability is assessed through the determination of a linear dominant dynamics, whose stability features imply the desired result similar to the development in [36,37,45].

Note that for all $p_{ki}\in \left[0,1\right]$, k={A,B}, i=1,…,N it holds that

$$\begin{array}{cc} q_{Ai} & =\prod_{j=1}^{N}\left(1-\beta_{Ai}a_{ij}p_{Aj}\right)\left(1-\gamma_{Ai}p_{Bi}\right)\\ \; & \geq \left(1-\sum_{j}\beta_{Ai}a_{ij}p_{Aj}\right)\left(1-\gamma_{Ai}p_{Bi}\right)\\ \; & =1-\sum_{j}\beta_{Ai}a_{ij}p_{Aj}-\gamma_{Ai}p_{Bi}+\sum_{j}\beta_{Ai}a_{ij}p_{Aj}\gamma_{Ai}p_{Bi} \end{array}$$

where in the second step the Weierstrass product inequality [49] has been employed. It follows that

$$\begin{array}{cc} 1-q_{Ai} & \leq \sum_{j}\beta_{Ai}a_{ij}p_{Aj}+\gamma_{Ai}p_{Bi}-\sum_{j}\beta_{Ai}a_{ij}p_{Aj}\gamma_{Ai}p_{Bi}\\ \; & \leq \sum_{j}\beta_{Ai}a_{ij}p_{Aj}+\gamma_{Ai}p_{Bi}. \end{array}$$

Equivalently it holds that

$$\begin{array}{cc} 1-q_{Bi} & \leq \sum_{j=1}^{N}\beta_{Bi}b_{ij}p_{Bj}+\gamma_{Bi}p_{Ai}. \end{array}$$

Substitution of these inequalities into Equations (1) and taking into account that $0\leq 1-p_{ki}\leq 1$ holds true it follows that

(6) $$\begin{array}{c} \begin{array}{cc} p_{Ai}\left(t+1\right) & \leq \left(1-\mu_{Ai}\right)p_{Ai}\left(t\right)+\sum_{j=1}^{N}\beta_{Ai}a_{ij}p_{Aj}\left(t\right)+\gamma_{Ai}p_{Bi}\left(t\right),\\ p_{Bi}\left(t+1\right) & \leq \gamma_{Bi}p_{Ai}\left(t\right)+\left(1-\mu_{Bi}\right)p_{Bi}\left(t\right)+\sum_{j=1}^{N}\beta_{Bi}b_{ij}p_{Bj}\left(t\right). \end{array} \end{array}$$

The preceding Equations can be written in matrix form as

(7) $$\left[\begin{array}{c} P_{A}\left(t+1\right)\\ P_{B}\left(t+1\right) \end{array}\right]\leq \left[\begin{array}{cc} I-M_{A}+B_{A}A & G_{A}\\ G_{B} & I-M_{B}+B_{B}B \end{array}\right]\left[\begin{array}{c} P_{A}\left(t\right)\\ P_{B}\left(t\right) \end{array}\right]\leq H\left[\begin{array}{c} P_{A}\left(t\right)\\ P_{B}\left(t\right) \end{array}\right]$$

with I, $M_{k},B_{k}$ and $G_{k},\;k=A,B$ defined in the statement of Lemma 1. In virtue of (5) it follows that there exists a constant α=σ(H)∈(0,1) so that

$$\begin{array}{c} ∥\left[\begin{array}{c} P_{A}\left(t+1\right)\\ P_{B}\left(t+1\right) \end{array}\right]∥<\alpha ∥\left[\begin{array}{c} P_{A}\left(t\right)\\ P_{B}\left(t\right) \end{array}\right]∥ \end{array}$$

implying the exponential stability (4) of the extinction state. □

**Remark** **1.** *It should be noted at this place that according to the dynamics in* (7) *for the asymptotic stability of the origin ${\left[P_{a}^{T},P_{B}^{T}\right]}^{T}=0$ it is not sufficient to ensure the asymptotic stability in both sub-networks, what would be ensured by analyzing the diagonal sub-matrices $I-M_{K}+B_{K}K$ separately for K=A,B, but that it is required to account explicitly for the particular interconnection structure and the associated transition probabilities between sub-networks. This establishes a significant difference to the case of single-layer networks as considered e.g., in [36,37,38]. Given that the solutions of the linear dynamics* (7) *bound the one for the nonlinear dynamics, Theorem 1 is intrinsically connected with the input-to-state stability and the small-gain condition [47,48] for the interconnection* (1).

## 4. Control Design

The next question to be addressed is how the sufficient condition established in Theorem 1 can be used to design an efficient control strategy, and how the number of nodes to be controlled varies when considering the interconnection of two networks. This question is addressed in the following Lemma.

**Lemma** **2.** Let $N_{ki}$, k={A,B}, i=1,…,N denote the number of neighbors of node i in network k. For constant values $\mu_{ki}$, $\gamma_{ki}^{*}$, and $\beta_{ki}^{*}$, the extinction state is (globally in $P={\left[0,1\right]}^{2N}$) exponentially stable if for every node i in A and B it holds that

(8a) $$\begin{array}{cc} \mu_{Ai} & >\gamma_{Ai}^{*}+\beta_{Ai}^{*}N_{Ai}, \end{array}$$

(8b) $$\begin{array}{cc} \mu_{Bi} & >\gamma_{Bi}^{*}+\beta_{Bi}^{*}N_{Bi}. \end{array}$$

**Proof.**  In virtue of Lemma 1, it is sufficient to show that the conditions (8) ensure that σ(H)<1. This is achieved by applying Geršgorin’s theorem [50] to the matrix H using an upper-bound estimate for the spectral radius.

Let λ be an arbitrary eigenvalue of H. Recalling that all entries of the matrices A and B are non-negative, Geršgorin’s theorem [50] implies the following inequalities

$$\begin{array}{cc} \left|\lambda \right| & \leq \gamma_{Ai}^{*}+\sum_{j=1}^{N}\beta_{Ai}^{*}a_{ij}+1-\mu_{Ai},\\ \left|\lambda \right| & \leq \gamma_{Bi}^{*}+\sum_{j=1}^{N}\beta_{Bi}^{*}b_{ij}+1-\mu_{Bi}. \end{array}$$

Thus |λ|<1 is satisfied if

$$\begin{array}{cc} \left|\lambda \right| & <\gamma_{Ai}^{*}+\sum_{j=1}^{N}\beta_{Ai}^{*}a_{ij}+1-\mu_{Ai}<1,\\ \left|\lambda \right| & <\gamma_{Bi}^{*}+\sum_{j=1}^{N}\beta_{Bi}^{*}b_{ij}+1-\mu_{Bi}<1. \end{array}$$

Rearranging and taking into account that the numbers of neighbors of node *i* in network A and B is given by $N_{Ai}=\sum_{j=1}^{N}a_{ij},N_{Bi}=\sum_{j=1}^{N}b_{ij}$, respectively, it follows that this condition is satisfied if

$$\begin{array}{cc} \gamma_{Ai}^{*}+\sum_{j=1}^{N}\beta_{Ai}^{*}a_{ij}=\gamma_{Ai}^{*}+\beta_{Ai}^{*}N_{Ai} & <\mu_{Ai},\\ \gamma_{Bi}^{*}+\sum_{j=1}^{N}\beta_{Bi}^{*}b_{ij}=\gamma_{Bi}^{*}+\beta_{Bi}^{*}N_{Bi} & <\mu_{Bi}, \end{array}$$

for i=1,2,…,N. These inequalities correspond to the ones stated in (8). □

**Remark** **2.** *The stability conditions* (8) *of the system basically state that the recovery rate of each node must be higher than the rate with which it potentially receives infected messages or has contact with infected neighbors, measured by the total amount of intra-layer contacts in each network k={A,B} during one time interval, i.e., $\beta_{ki}N_{ki}$ plus the inter-layer contacts $\gamma_{ki}$ during the same time interval.*

Condition (8) can be used to determine which nodes should be controlled, i.e., for which nodes *i* inequalities (8) are not satisfied in either of the networks *A* and/ or *B* and thus either of the rates $\gamma_{ki}$ or $\beta_{ki}$ should be adapted in such a way that $\gamma_{ki}<\gamma_{ki}^{*}$ and/ or $\beta_{ki}<\beta_{ki}^{*}$ with $\gamma_{ki}^{*},\beta_{ki}^{*}$ chosen so that (8) holds. This is summarized in the following corollary.

**Corollary** **1.** *The extinction state is (globally in P) exponentially stable if for all nodes i for which either of the conditions in* (8) *does not hold the parameter $\gamma_{ki}$ and/ or $\beta_{ki}$ are adapted so that the inequalities* (8) *are satisfied.*

**Remark** **3.** *It should be noted that the conditions of Corollary 1 are only sufficient and not necessary. Actually, in specific scenarios the number of nodes for which the transmission parameters have to be adapted can be smaller. Alternative (non-analytic) approaches to determine the nodes to be controlled would be e.g., using optimization or genetic algorithms.*

**Remark** **4.** *In comparison with the single-layer setup considered, e.g., in [36,37,38] the additional dependency on $\gamma_{ki},\;k=A,B$ introduces stronger conditions. This will most probably imply a higher number of nodes to be controlled in the case of interconnecting the network with another one, i.e., the number of nodes that need to be controlled to ensure an asymptotically stable interconnection will be larger then the sum of the numbers of nodes that need to be controlled in each sub-network to achieve individual asymptotic stability. This is a particularly important point highlighting a consequence of the complex interplay of two nonlinear dynamical systems pointed out in Remark 1.*

**Remark** **5.** *Conditions* (8), *as alternative to Corollary 1, also suggests as sufficient condition, to adapt the parameters $N_{Ai}$ and/or $N_{Bi}$. This adaptation requires disconnecting links from those nodes that do not satisfy condition* (8) *in order to reach the extinction state, resulting in an equivalent method as the one proposed in Adaptive Networks [39,46]. However, our approach keeps the network structure, modifying the parameters associated with the interaction probabilities of the model, avoiding disconnecting nodes.*

According to inequalities (8), a set of all possible scenarios for adaptation of parameters in every layer and for every node is presented in Table 1. That means that every node could have a different set of parameter to be controlled as shown in the Table, with the exception of those nodes that satisfied the condition (8) that do not need to be controlled as is shown in scenario 1. We can notice that in scenario 2 the critical parameter (i.e., the parameter to be controlled) of node *i*, situated in layer k={A,B}, is given by $\gamma_{ki}$. For the scenario 5 we have several options and the criterion to be selected will depend on the specific implementation costs varying with the particular case example at hand.

Note from Table 1 that it is not necessary for the nodes of any layer to be acquainted of the structure and properties of the nodes of the other layer in order to control and eventually reach the extinction state. This constitutes one of the virtues of non centralized control.

## 5. Simulations

To corroborate the theoretical results, numerical simulations have been performed considering a spreading process in a two-layer network with $N=10^{5}$ nodes in each layer. In the simulations performed, in order to verify that our results are independent of the topology, we have selected three different types of networks: Barabási–Albert scale-free (BA type), Regular nearest-neighbor (R type) and Small-World (WS type). Every network was built according to the methods discussed in [51], and as it is stated in this reference, the WS network was constructed randomly rewiring a Regular network with parameters shown in Table 2. As stated above, each node in layer *A* is connected to its counterpart in layer *B*.

For the subsequent analysis the parameter intervals shown in Table 3 were selected for $\mu_{i}$, $\gamma_{i}$ and $\beta_{i}$ and every type of network in Table 2 and for every node i=1,2,…,N in such a way that a considerable endemic response can be observed when the network parameters are uniformly distributed over these intervals.

Considering the parameters shown in Table 2, six network layers were built (two networks for each network BA, *R* and WS) that were combined to form six different two-layer networks as listed in Table 4. The parameters of each node in each layer were assigned randomly according to the intervals given in Table 3. Based on these scenarios the nodes to be controlled were identified and classified according to Table 1 to establish a control criteria. The results are summarized in Table 5 showing the number of nodes for which γ needs to be adjusted, those for which β needs to be adjusted, those for which either of both needs to be adjusted and those for which both need to be adjusted. Accordingly, the total number of nodes to be controlled is given in the last column.

The difference between analyzing and controlling the networks in a single layer context to the two-layer one becomes clear when comparing the numbers in Table 4. Without interconnection of the two layers only the third column is relevant, i.e., the number of nodes for which β must be adjusted. It can be clearly seen that due to the coupling with a second layer very drastic changes occur, independent of the choice of topology in the attached layer. In particular, consider an interconnection of $R_{1}$ and $WS_{1}$. In the isolated network $R_{1}$ no node needs to be controlled as the extinction point is globally asymptotically stable. The network $WS_{1}$, when isolated only requires 30 nodes to be controlled. When interconnecting both networks it becomes necessary to control 87,387 nodes in $R_{1}$ and 86,470 in $WS_{1}$.

Note further that according to Table 5 several scenarios could arise depending on the networks selected to build the two layer multiplex network, for example, if we propose a two layer multiplex network made up of $R_{2}$ (layer *A*) and $BA_{2}$ (layer *B*) then, according to Table 5, it is only necessary to control both networks taking β as amenable parameter.

In order to show the effect of the proposed control law, we simulate several two layer networks as described in Table 4. The changes in the transmission parameters are applied at time 35. In these simulations, and following the above discussion, the specific values for the control parameters are chosen either as one of the following:

(9a) $$\begin{array}{ccc} \beta_{ki}\left(t\right) & = & \left\{\begin{array}{cc} \beta_{ki}\; & t<35,\\ 0.99\frac{\mu ki-\gamma ki}{N_{ki}}\; & t\geq 35 \end{array}\right. \end{array}$$

(9b) $$\begin{array}{ccc} \gamma_{ki}\left(t\right) & = & \left\{\begin{array}{cc} \gamma_{ki}\; & t<35,\\ 0.99(\mu ki-\beta kiN_{ki})\; & t\geq 35 \end{array}\right. \end{array}$$

for k={A,B} and i=1,2,…,N. Besides, in this case it is also possible to chose γ and β (at the same time) as control parameters (scenario 4 from Table 1). This is also the case of networks 2, 3 and 5 in Table 4, where an specific combination of control parameters are chosen as $\gamma_{ki}=0.99\mu_{ki}$ and (9a).

**Figure 2:**
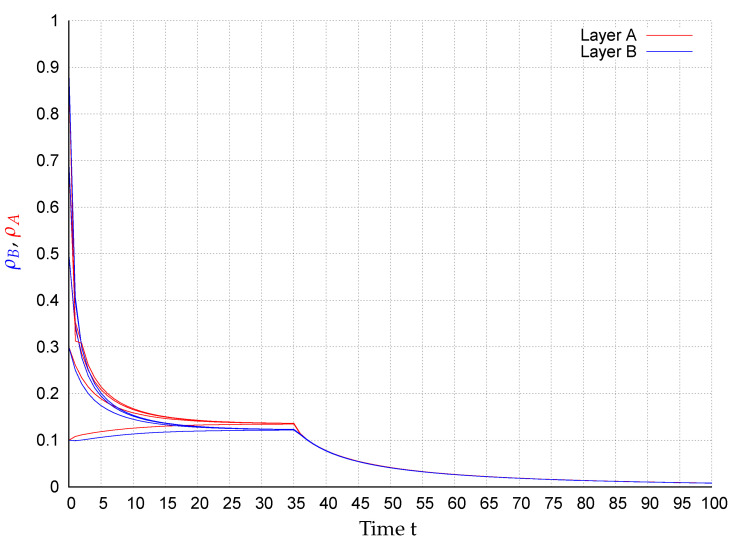
$\rho_{A}\left(t\right)$ (red) and $\rho_{B}\left(t\right)$ (blue) for several initial conditions in network $R_{1}$-$R_{2}$.

**Figure 3:**
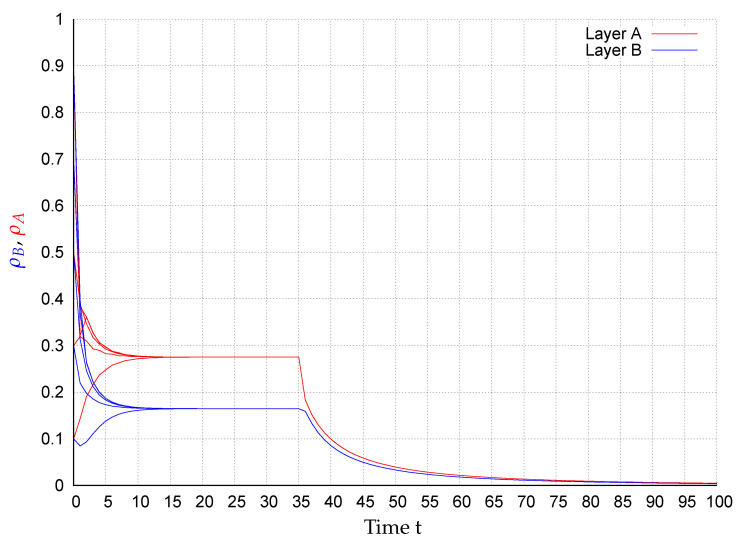
$\rho_{A}\left(t\right)$ (red) and $\rho_{B}\left(t\right)$ (blue) for several initial conditions in network $BA_{1}$-$BA_{2}$.

**Figure 4:**
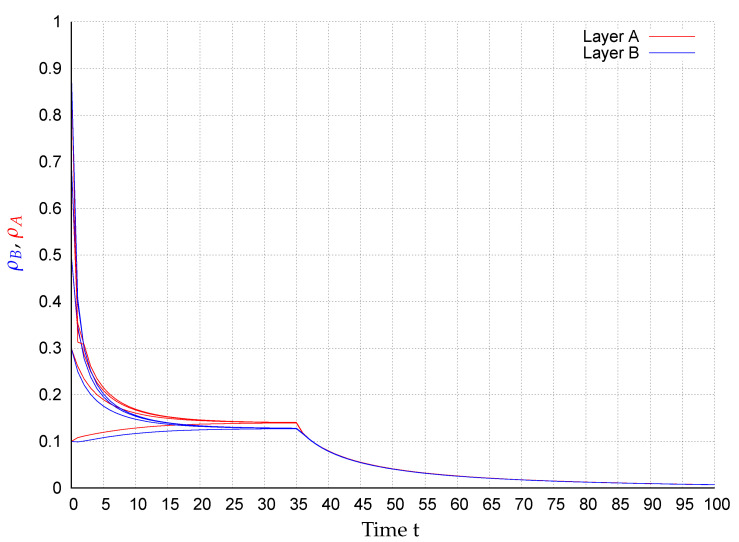
$\rho_{A}\left(t\right)$ (red) and $\rho_{B}\left(t\right)$ (blue) for several initial conditions in network $WS_{1}$-$WS_{2}$.

**Figure 5:**
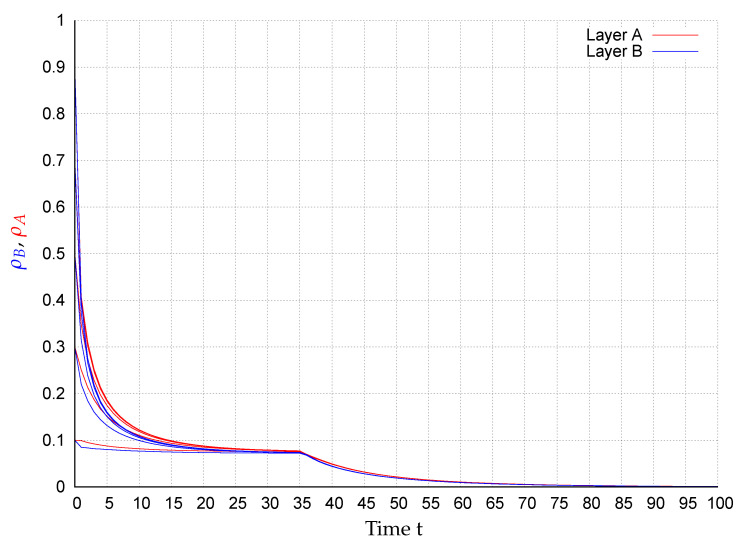
$\rho_{A}\left(t\right)$ (red) and $\rho_{B}\left(t\right)$ (blue) for several initial conditions in network $R_{2}$-$BA_{2}$.

**Figure 6:**
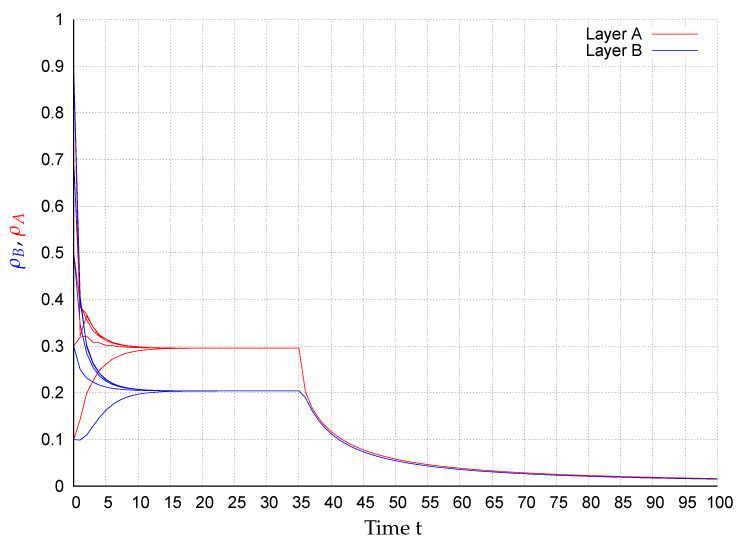
$\rho_{A}\left(t\right)$ (red) and $\rho_{B}\left(t\right)$ (blue) for several initial conditions in network $BA_{1}$-$WS_{2}$.

**Figure 7:**
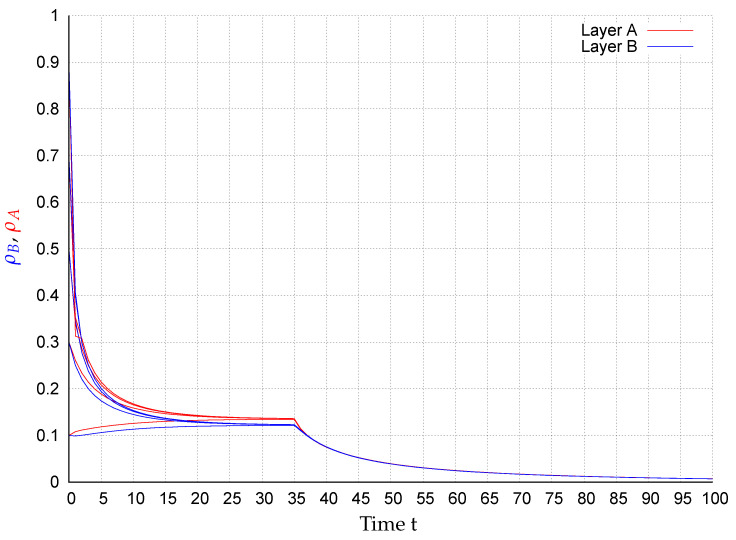
$\rho_{A}\left(t\right)$ (red) and $\rho_{B}\left(t\right)$ (blue) for several initial conditions in network $R_{1}$-$WS_{2}$.

In consequence of this control scenario, at the beginning the state converge to an endemic fixed point that disappears after applying the control strategy at t=35, causing the states to exponentially converge to the extinction state, as shown in Figure 2, Figure 3, Figure 4, Figure 5, Figure 6 and Figure 7. In the figures each line corresponds to the mean value (or probability density)

(10) $$\rho_{A}\left(t\right)=\sum_{i=1}^{N}p_{Ai}\left(t\right)\;(\mathrm{red})\mathrm{and}\;\rho_{B}\left(t\right)=\sum_{i=1}^{N}p_{Bi}\left(t\right)\;\left(\mathrm{blue}\right),$$

in the respective network for the initial conditions $p_{Ai}\left(0\right),p_{Bi}\left(0\right)\in \left\{0.1,0.3,0.5,0.7,0.9\right\},\;i=1,\ldots ,N$. For example, in Figure 3 around 28% of the nodes in layer *A* are infected meanwhile in network *B*, around 17% of the nodes are. Once the control is activated, in all simulations, the state of the system exponentially converges to the extinction state according to the assertion of Corollary 1.

**Figure 8:**
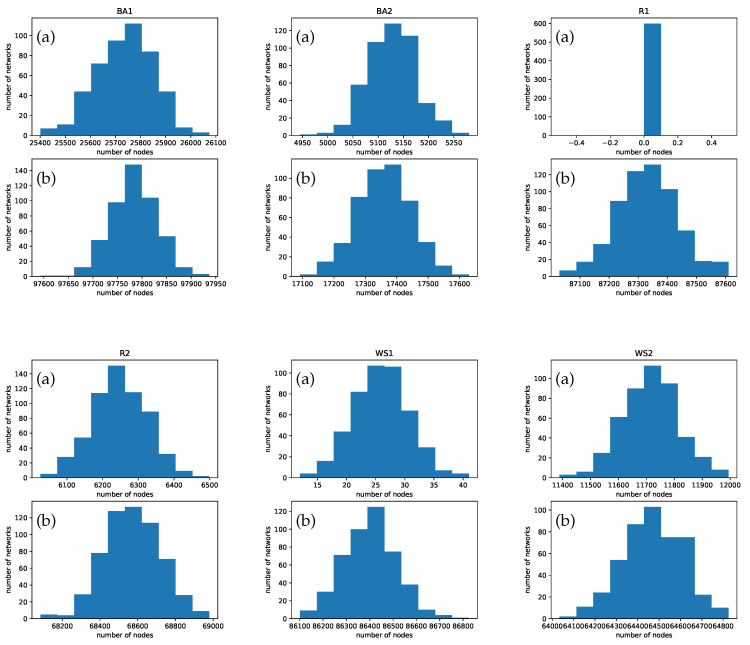
Sample distributions of the number of nodes to be controlled in the considered networks specified in Table 2: (**a**) isolated network and (**b**) interconnected network.

In order to analyze the dependency of the number of nodes to be controlled on the particular choice of network a statistical analysis has been carried out for the networks $BA_{1},BA_{2},R_{1},R_{2},WS_{1},WS_{2}$ with construction specified in Table 2 by randomly assigning the seeds for the network generation and the parameters using a uniform distribution over the intervals provided in Table 3. For the BA-type networks a total of 481 networks were considered, for the *R*-type networks 600, and for the WS-type networks 464. The resulting sample distributions showing the number of times a certain number of nodes needs to be controlled are shown in Figure 8. For all six networks two scenarios are evaluated: (a) the isolated network and (b) the network in interconnection with another one. From the sub-figures it can be seen that (i) in all networks a very small variation is observed in the number of nodes to be controlled, and (ii) in the passage from the isolated to the interconnected network the number of nodes to be controlled increases considerably. This last fact illustrates again the substantial difference between controlling isolated and interconnected networks, as highlighted above at several places.

## 6. Conclusions

The control of a spreading process in a two-layer multiplex network with a parametric control strategy is analyzed. Sufficient conditions for the choice of nodes and parameters to be controlled are established using rigorous mathematical derivations ensuring the exponential stability of the extinction state globally with respect to the set of all possible probability states. The proposed control strategy consists in the adaptation of the parameters specifying the intra-layer and inter-layer transmission rates only for a limited number of nodes that are characterized by a parametric threshold condition. Particular emphasis is made on the substantial difference between controlling isolated and interconnected networks, showing intrinsic cnections with the individual input-to-state stability and the small-gain criterion. It results that in the passage from controlling isolated networks to interconnected ones, the number of nodes that need to be controlled significantly increases. The theoretical results are analyzed in multiplex networks with different representative topologies in each layer with $10^{5}$ nodes each. The corresponding simulation studies and statistical evaluations of the number of nodes to be controlled corroborate the theoretical findings.

Based on the presented results future studies will focus on the generalization of the discussed ideas to the case of *n*-layer multiplex networks, in order to further enlighten the expected challenges when adding additional layers. Furthermore, the model identification and testing of the presented approaches in real-world scenarios based on explicit data will be focused on in future studies.

## Figures and Tables

**Table 1:** Amenable control parameters for the nodes of every layer k={A,B}.

Scenario	Critical Parameter	Satisfied	Not Satisfied
1	-	$\mu_{ki}>\gamma_{ki}+\beta_{ki}N_{ki}$	-
2	$\gamma_{ki}$	$\mu_{ki}-\beta_{ki}N_{ki}\geq 0$	$\mu_{ki}>\gamma_{ki}+\beta_{ki}N_{ki}$
3	$\beta_{ki}$	$\mu_{ki}-\gamma_{ki}\geq 0$	$\mu_{ki}>\gamma_{ki}+\beta_{ki}N_{ki}$
4	$\gamma_{ki}$ and $\beta_{ki}$	-	$\mu_{ki}>\gamma_{ki}+\beta_{ki}N_{ki}$
5	$\gamma_{ki}$ or $\beta_{ki}$	$\mu_{ki}-\beta_{ki}N_{ki}\geq 0$	$\mu_{ki}>\gamma_{ki}+\beta_{ki}N_{ki}$
		$\mu_{ki}-\gamma_{ki}\geq 0$	

**Table 2:** Construction parameters for networks Barábasi-Albert (BA), Regular (*R*) and Small-World (WS).

Network	Parameters
$BA_{1}$	$m_{0}=10$, m=2
$BA_{2}$	$m_{0}=5$, m=3
$R_{1}$	Every node is connected with 20 nearest neighbors.
$R_{2}$	Every node is connected with 10 nearest neighbors.
$WS_{1}$	Every node in $R_{1}$ network was randomly rewired with probability 0.2.
$WS_{2}$	Every node in $R_{2}$ network was randomly rewired with probability 0.3.

**Table 3:** Simulation parameters for each node i=1,2,…,N, in every network in Table 2.

Network	$\mu_{i}$	$\gamma_{i}$	$\beta_{i}$
$BA_{1}$, $R_{1}$, $WS_{1}$	(0.60,0.80)	(0.40,0.80)	(0.01,0.03)
$BA_{2}$, $R_{2}$, $WS_{2}$	(0.50,0.70)	(0.20,0.35)	(0.02,0.06)

**Table 4:** Amenable parameters chosen to control every two layer network. Compare this with data shown in Table 5.

No.	Layer *A*	Layer *B*	Amenable Parameters Chosen	Figure
1	$R_{1}$	$R_{2}$	$\beta_{Ai}$ and $\beta_{Bi}$	2
2	$BA_{1}$	$BA_{2}$	$\gamma_{Ai}$, $\beta_{Ai}$ and $\beta_{Bi}$	3
3	$WS_{1}$	$WS_{2}$	$\gamma_{Ai}$, $\beta_{Ai}$ and $\beta_{Bi}$	4
4	$R_{2}$	$BA_{2}$	$\beta_{Ai}$ and $\beta_{Bi}$	5
5	$BA_{1}$	$WS_{2}$	$\gamma_{Ai}$, $\beta_{Ai}$ and $\beta_{Bi}$	6
6	$R_{1}$	$WS_{2}$	$\gamma_{Ai}$ and $\beta_{Bi}$	7

**Table 5:** Number of nodes and their parameters to control for every network.

Network	$\gamma_{i}$	$\beta_{i}$	$\gamma_{i}$ or $\beta_{i}$	$\gamma_{i}$ and $\beta_{i}$	Nodes to Control
$BA_{1}$	12,178	25,716	52,080	7761	97,735
$BA_{2}$	0	5082	12,191	0	17,273
$R_{1}$	19,939	0	67,448	0	87,387
$R_{2}$	0	6138	62,286	0	68,424
$WS_{1}$	19,915	30	66,501	24	86,470
$WS_{2}$	0	11,697	52,678	0	64,375

## Abbreviations

The following abbreviations are used in this manuscript:

BA	Barabási-Albert scale free network
R	Regular nearest-neighbor network
WS	Watts-Strogatz small-world network
MDPI	Multidisciplinary Digital Publishing Institute
